# Mast cell driven immunometabolism as a therapeutic entry point in ESCC

**DOI:** 10.3389/fcell.2026.1830394

**Published:** 2026-05-11

**Authors:** Zhifeng Qu, Xuewei Zheng, Anshun Zhao, Pei Wang, Shegan Gao, Qinan Yin

**Affiliations:** 1 Department of Radiation Oncology, Cancer Institute, The First Affiliated Hospital, and College of Clinical Medicine of Henan University of Science and Technology, Luoyang, Henan, China; 2 State Key Laboratory of Natural and Biomimetic Drugs, Peking University, Beijing, China; 3 Precision Medicine Laboratory, School of Medical Technology and Engineering, Henan University of Science and Technology, Luoyang, Henan, China; 4 Henan Key Laboratory of Microbiome and Esophageal Cancer Prevention and Treatment, Henan Key Laboratory of Cancer Epigenetics, The First Affiliated Hospital of Henan University of Science and Technology, Luoyang, Henan, China

**Keywords:** esophageal squamous cell carcinoma, immunometabolism, immunotherapy response, lipid metabolism, mast cells, tumor microenvironment

## Abstract

Esophageal squamous cell carcinoma (ESCC) remains one of the most aggressive epithelial malignancies, with most patients deriving only modest benefit from surgery, chemoradiotherapy, or immune checkpoint inhibition. Recent studies suggest that tumor metabolic reprogramming and immune dysfunction evolve together and reinforce one another, yet the causal links between these processes remain only partially understood. Mast cells (MCs) represent a stromal population that has received more attention. Although associated with allergic reactions, tissue repair, and inflammatory responses under normal physiology, MCs in ESCC frequently occupy stromal, vascular and hypoxic zones where metabolic stress is most pronounced. This spatial distribution suggests that MCs actively shape tumor metabolic states through the release of lipid mediators, including prostaglandins, leukotrienes, and platelet activating factor (PAF). These mediators amplify lipid metabolic programs in tumor cells and contribute to an immunosuppressive environment in which dendritic cell priming is attenuated, and cytotoxic T cell (CTL) function is progressively impaired. MC-derived cytokines and proteases further remodel the extracellular matrix and reorganize stromal architecture, collectively facilitating the invasion of malignant cells into adjacent tissue. Single cell and spatial transcriptomic analyses have revealed substantial heterogeneity among tumor-infiltrating MCs, indicating that distinct phenotypic subsets engage divergent metabolic and immune circuits and that only a subset may be functionally tumor permissive. These findings have generated increasing interest in therapeutic strategies targeting MC-linked mediators, inhibiting lipid metabolic enzymes, or integrating metabolic modulation with immune checkpoint therapy. A rigorous mechanistic understanding of how MCs coordinate metabolic and immune remodeling in ESCC may ultimately support biomarker-guided patient stratification and inform novel therapeutic combinations capable of overcoming resistance to current treatment modalities.

## 1 Introduction

ESCC represents a formidable global health challenge, particularly across East Asia and Africa (Sung et al., 2021). Despite the integration of advanced surgical techniques, chemoradiotherapy, and immunotherapy, the overall prognosis remains suboptimal due to rapid biological progression and intrinsic therapeutic resistance (Pennathur et al., 2013; Rustgi and El-Serag, 2014; Lin et al., 2018; Abnet et al., 2018).

Metabolic reprogramming is now recognized as a fundamental hallmark of this malignancy. Cancer cells execute comprehensive rewiring of metabolic pathways to sustain proliferation and modulate the surrounding microenvironment (Hanahan, 2022; Pavlova and Thompson, 2016). In ESCC, profound alterations in lipid metabolism, including accelerated arachidonic acid signaling and deregulated fatty acid synthesis, serve as primary drivers of tumor invasion and immune evasion (Cui et al., 2022; Jiao et al., 2024). Recent multi-omic characterizations further confirm that these lipid metabolic signatures are functionally coupled with immune dysfunction and clinical refractory states (Lin et al., 2024).

The ESCC immune landscape is defined by a suppressive consortium of regulatory T cells (Tregs), myeloid-derived suppressor cells (MDSCs), and M2-polarized macrophages (Zheng et al., 2020). These populations cooperate with metabolic byproducts such as prostaglandin E2 (PGE2) to impair dendritic cell maturation and drive the terminal exhaustion of cytotoxic lymphocytes (Cui et al., 2022; Liu et al., 2025). Collectively, this interdependence suggests that ESCC progression is governed by a synchronized process of immunometabolic remodeling rather than isolated events. Within this ecosystem, MCs represent a pivotal yet incompletely characterized cellular compartment. While historically categorized as effector cells of allergic inflammation, intratumoral MCs are now recognized as potent modulators of cancer biology (de Visser and Joyce, 2023).

Through the regulated secretion of cytokines, proteases, and bioactive lipid mediators, MCs influence neoangiogenesis, stromal remodeling, and leukocyte activation (de Visser and Joyce, 2023; Segura-Villalobos et al., 2022; Özdemir et al., 2024; Sattiraju et al., 2023). Their strategic localization within perivascular and hypoxic niches suggests a sentinel function that transduces metabolic cues into sustained inflammatory or suppressive signals. Although MC-derived eicosanoids and PAF promote suppressive immune circuits in various malignancies, their specific metabolic phenotypes and lineage-specific functional programs in ESCC remain poorly defined (Cui et al., 2022; Wculek and Malanchi, 2015). Preliminary evidence correlating MC infiltration with adverse clinical outcomes underscores the necessity of delineating their mechanistic contribution (Zheng et al., 2020).

This review synthesizes current evidence to propose a mechanistic framework in which MCs serve as active organizers of ESCC immunometabolic remodeling, with the goal of identifying actionable targets for biomarker-based intervention.

## 2 Biology and roles of MCs in cancer

MCs are tissue-resident immune cells originating from hematopoietic progenitors and undergoing terminal maturation within peripheral tissues (Lichterman and Reddy, 2021; Moreira et al., 2018). They are defined by expression of FcεRI and the receptor tyrosine kinase KIT/CD117, which together regulate their activation, survival, and secretory programs (Krystel-Whittemore et al., 2015; Okayama and Kawakami, 2006). Upon stimulation, MCs release pre-formed granules containing mediators such as histamine, tryptase, and chymase, and subsequently synthesize cytokines, chemokines, and lipid-derived mediators that orchestrate immune cell recruitment, vascular dynamics, and stromal remodeling (Mukai et al., 2018; Oskeritzian, 2012; Ud-Din et al., 2020).

While MCs can exhibit antitumor effects, in most solid cancers their activity supports tumor growth through vascular expansion, stromal remodeling, and establishing immunosuppressive immune circuits (Segura-Villalobos et al., 2022). Their secretome includes vascular endothelial growth factor (VEGF), fibroblast growth factor-2 (FGF-2), interleukin-10 (IL-10) and transforming growth factor-β (TGF-β), and matrix-metalloproteinases which together assist tumor invasion and metastatic spread (Segura-Villalobos et al., 2022; Lichterman and Reddy, 2021). MCs also influence tumor metabolic adaptation by releasing lipid mediators such as prostaglandins, leukotrienes, and PAF, which affect metabolic pathways in tumor cells and immune infiltrates (Pandey et al., 2024). PGE2 can enhance fatty-acid synthesis and glycolysis while weakening antigen presentation and cytotoxic lymphocyte activity, positioning MCs at the interface of metabolic and immune reprogramming (Bayerl et al., 2023; Pascual and Benitah, 2024).

Single-cell RNA sequencing (scRNA-seq) and spatial transcriptomic studies reveal transcriptional heterogeneity among tumor-infiltrating MCs, including clusters enriched in lipid metabolic signatures, hypoxia-responsive programs, and immunosuppressive cytokines (Zhang D. et al., 2024; Chang et al., 2025; Li et al., 2024). These findings suggest MCs comprise functionally specialized subgroups that engage metabolic and immune circuits in a subset dependent manner. Although direct evidence in ESCC is limited, immunohistochemical studies show MC localization near vasculature and invasive fronts in ESCC tissues, associated with worse prognosis. Given the dysregulated lipid landscape and highly immunosuppressive ESCC microenvironment, functional studies are needed to define MC subtypes, map their metabolic secretome, and evaluate whether targeting MC-derived lipid mediators can augment therapeutic efficacy in ESCC.

## 3 Lipid metabolic reprogramming in ESCC

Lipid metabolic dysregulation is a defining alteration in ESCC and is increasingly recognized as a functional driver rather than a secondary metabolic consequence (Wang et al., 2024a). Multi-omics analyses have identified lipid signatures that stratify clinical prognosis and overlap with immune-excluded phenotypes, supporting the concept that lipid metabolism and immune remodeling are tightly interconnected in ESCC (Guo Z-X. et al., 2025; Sun et al., 2025).

### 3.1 Arachidonic acid pathway and the cyclooxygenase-2 (COX-2)/PGE2 axis

Arachidonic-acid metabolism undergoes profound reprogramming in ESCC, most notably reflected by consistent overexpression of COX-2 and elevated levels of PGE2 within tumor tissues (Jiang et al., 2004; Moon et al., 2020). COX-2 catalyzes the conversion of arachidonic acid to PGE2, a pleiotropic mediator that drives tumor proliferation, angiogenesis, epithelial-mesenchymal transition (EMT), and the recruitment of immunosuppressive populations including Tregs and MDSCs (Guo et al., 2018; Cui et al., 2015; Wilson and DuBois, 2022). PGE2 signaling further contributes to immune escape through induction of programmed death-ligand 1 (PD-L1) expression on tumor and infiltrating immune cells, and has been associated with chemoresistance and inferior clinical outcomes in ESCC (Wilson and DuBois, 2022).

Transcriptomic profiling further implicates EP4-dependent PGE2 signaling as a dominant suppressor of CD8^+^ T cell infiltration and cytolytic capacity, supporting the concept that arachidonic-acid metabolism directly shapes immune dysfunction in ESCC (Cuenca-Escalona et al., 2024). Nevertheless, the relative contribution of tumor-intrinsic versus stromal sources of PGE2 remains unresolved and represents a key mechanistic gap. Addressing this question has direct translational relevance, as selective inhibition of COX-2 or downstream EP4 receptors is being evaluated as a strategy to enhance checkpoint blockade efficacy and overcome immune resistance mediated by this signaling axis (Santiso et al., 2024). Within the ESCC stroma, MCs represent a major source of PGE2 and are positioned to amplify PGE2-driven metabolic suppression and maintain an immune-restricted microenvironment (Nie et al., 2023).

### 3.2 Fatty-acid synthesis machinery and lipid anabolism in ESCC

In addition to arachidonic acid metabolism, phospholipid remodeling and fatty acid synthesis are significantly dysregulated in ESCC (Tao et al., 2021). Fatty acid synthase (FASN), a key enzyme responsible for *de novo* fatty acid synthesis, is frequently overexpressed in ESCC tissues (Orita et al., 2010). FASN-driven lipid synthesis supplies critical components for membrane biogenesis, energy storage, and signaling lipid production, all of which are essential for sustaining rapid tumor cell proliferation (Du et al., 2023). Moreover, FASN activity has been shown to activate oncogenic pathways such as phosphoinositide-3 kinase/protein kinase-B/mammalian target of rapamycin (PI3K/Akt/mTOR) pathway signaling, further promoting tumor survival and progression (Shi J-j et al., 2019). Multi-omics profiling has identified sterol regulatory element-binding protein 1 (SREBP1) overexpression as a biomarker of metastatic ESCC and suggests that lipogenic reprogramming is closely aligned with early recurrence (Li et al., 2021). Blocking fatty acid synthesis partially restores antigen-presentation pathways, raising the possibility that ESCC invasion and immune suppression share metabolic determinants (Wang et al., 2024a). Complementing these tumor-intrinsic mechanisms, stromal biology evidence suggests that MC-derived PGE2 and LTB4 trigger a paracrine circuit that augments SREBP1 activity in fibroblasts, thereby reinforcing the lipogenic program within the ESCC microenvironment.

### 3.3 Cholesterol and sphingolipid-based rewiring

Aberrant cholesterol metabolism is a defining feature of ESCC, driven by SREBP1 signaling, which promotes transcription of acetyl-CoA carboxylase (ACC), FASN, and 3-hydroxy-3-methylglutaryl-CoA reductase (HMGCR), thereby sustaining membrane cholesterol enrichment, lipid-raft-dependent oncogenic signaling, and correlation with aggressive clinical phenotypes (Li et al., 2021; Zhao H. et al., 2023; Zhao et al., 2022). Beyond its intrinsic role in tumor cell survival, cholesterol metabolism functions as a mediator of immune evasion by impairing antigen presentation and suppressing the effector potency of cytotoxic T lymphocytes (Lagunas-Rangel, 2025). Transcriptomic analyses show that cholesterol-rich ESCC niches respond poorly to PD-1 blockade, indicating that metabolic rewiring modulates immunotherapy efficacy (Ko et al., 2024). Stromal MCs have been shown to release lipid-enriched extracellular vesicles containing sphingolipids, providing a potential mechanism by which stromal-tumor crosstalk sustains cholesterol-dependent signaling, although ESCC-specific validation remains elusive.

### 3.4 Integration of lipid metabolism with immune-escape circuits

Lipid metabolic reprogramming fundamentally impacts ESCC progression at multiple hierarchical levels. Enhanced fatty acid synthesis and lipid uptake support critical bioenergetic and biosynthetic requirements under hypoxic and nutrient limited TME conditions (Tao et al., 2021). Intracellular lipid droplets accumulate as metabolic reservoirs during cellular stress (Jin et al., 2023). Bioactive lipid-derived metabolites, including lysophosphatidic acid (LPA) and sphingosine-1-phosphate (S1P), activate ubiquitous pro-survival and pro-migratory signaling cascades (Lagunas-Rangel, 2025). Beyond these tumor-intrinsic effects, altered lipid metabolism shapes the immune microenvironment by generating potent immunosuppressive mediators and driving metabolic competition for essential nutrients between tumor cells and infiltrating T lymphocytes (Jin et al., 2023; Ma et al., 2019; Zhang GC. et al., 2023). Recent spatial immune profiling in ESCC has revealed a closed immunometabolic loop wherein the accumulation of PGE2 and cholesterol correlates with diminished CD8^+^ T cell infiltration, suggesting that metabolic remodeling actively enforces immune sequestration and facilitates occult metastatic progression (Liu et al., 2025).

These findings justify the exploration of combinatorial strategies involving metabolic inhibition and immune checkpoint blockade to abrogate ESCC-associated immune resistance. Given that MCs constitute a primary stromal reservoir of PGE2, leukotrienes, and PAF, they may function as upstream cellular initiators of lipid-mediated immunosuppression. This hypothesis remains to be experimentally validated and represents a critical frontier for future mechanistic investigation. An overview of MC–associated immunometabolic mechanisms in ESCC is summarized in Table 1.

**TABLE 1:** Mechanistic integration of MC-driven lipid metabolism and immune evasion in ESCC.

Mechanistic category	Key mediators or pathways	Functional consequences in ESCC	ESCC-specific evidence	References
Arachidonic acid derived signaling	COX-2, PGE2, EP4	Suppresses APC maturation and CD8^+^ T cell activation while promoting angiogenesis and EMT	COX-2 and PGE2 elevation correlate with EP4 dependent suppression of CD8^+^ cytolysis	Jiang et al. (2004), Cui et al. (2015), Wilson and DuBois (2022), Cuenca-Escalona et al. (2024), Santiso et al. (2024), Nie et al. (2023), Guo et al. (2025b)
Lipid anabolism and fatty acid synthesis	FASN, SREBP1, PI3K/Akt/mTOR	Facilitates membrane biogenesis and tumor survival while driving lipogenic recurrence	SREBP1 overexpression in metastatic ESCC and FASN linked restoration of antigen presentation	Orita et al. (2010), Du et al. (2023), Shi et al. (2019a), Li et al. (2021), Zhao et al. (2021)
Cholesterol and sphingolipid modulation	HMGCR, Cholesterol, Sphingolipid vesicles	Regulates lipid raft signaling and impairs CTL metabolic fitness to facilitate immune escape	Cholesterol- enriched niches correlate with attenuated response to PD-1 blockade	Zhao et al. (2023a), Lagunas-Rangel (2025), Zhao et al. (2021)
MC lipid mediator rewiring	MC-derived PGE2, LTB4, CysLTs, PAF/PAFR	Orchestrates stromal remodeling and EMT to restrict cytotoxic immune infiltration	PAFR expression in ESCC epithelium and MC eicosanoid mediated immunosuppression	Moore and Pidgeon (2017), Zhang et al. (2023b), Qu et al. (2025), Zhao et al. (2024)
MC cytokine driven modulation	IL-10, TGF-β, VEGF, Histamine	Impairs dendritic cell function and promotes Treg induction to shift Th1/Th2 equilibrium	Immunosuppressive MC signatures identified in ESCC spatial and cohort characterizations	Nie et al. (2023) ; Qu et al. (2025), Zhao et al. (2024) ; Komi and Redegeld (2020); Portales-Cervantes et al. (2019); Sreeramkumar et al. (2012); Shi Z. et al. (2019); Cui et al. (2017) ; Zhao D. et al. (2023) ; Xue et al. (2020)
MC metabolic phenotype	FASN, ACACA expression in MCs	Maintains sustained mediator secretion under metabolic stress in the tumor microenvironment (TME)	Proposed via metabolic profiling with ESCC specific validation currently pending	Mendoza et al. (2021), Wang et al. (2024b)

Recent advances in metabolomics and lipidomics have provided increasingly detailed evidence of the metabolic reprogramming landscape in ESCC, with direct implications for understanding how MCs operate within this biochemically reconfigured microenvironment. Lipidomic profiling studies have identified the accumulation of bioactive lipid species, including phospholipids, sphingolipids, and eicosanoids, which are closely associated with tumor progression and immune suppression (Sun et al., 2025; Si et al., 2024). Notably, elevated concentrations of arachidonic acid derived metabolites and cholesterol esters correlate with impaired cytotoxic T cell function and diminished antigen presentation capacity. This relationship underscores a functional link between dysregulated lipid metabolism and immune evasion. These lipidomic signatures represent active metabolic pathways rather than merely descriptive biomarkers. Specifically, phospholipase A2 mediates the liberation of arachidonic acid from membrane phosphatidylcholine and phosphatidylethanolamine pools. Subsequent oxygenation occurs via COX-2 to generate PGE2 and thromboxane A2 or via 5-lipoxygenase to produce LTB4 and cysteinyl leukotrienes. These enzymatic processes collectively determine the immunosuppressive potency of the ESCC lipid milieu (Jiang et al., 2004; Moore and Pidgeon, 2017). These same enzymatic routes are constitutively primed and can be rapidly deployed upon degranulation, making MCs uniquely positioned to amplify the arachidonic acid-derived immunosuppressive signals already present in the lipidomic landscape of ESCC tissue.

Building on this lipidomic framework, a comprehensive metabolic pathway-based stratification of ESCC using transcriptomic data from multiple independent cohorts has identified three molecularly distinct metabolic subtypes. These groups consist of the lipid dominant MPC1, the amino acid dominant MPC2, and the energy metabolism dominant MPC3 (Wang et al., 2024c). LC-MS-based metabolite profiling confirmed that MPC1 cell lines are characterized by a pronounced accumulation of triacylglycerols and complex glycerophospholipids. This profile is consistent with enhanced FASN-driven *de novo* lipogenesis and the preferential storage of esterified fatty acids. By contrast, MPC3 cell lines show a marked elevation of acylcarnitines which serve as the obligate intermediates for mitochondrial fatty acid import and beta oxidation. This elevation indicates a metabolic shift toward lipid catabolism as the primary energy strategy (Wang et al., 2024c). Critically, MPC3 was associated with the poorest clinical response to PD-L1 immunotherapy among the three subtypes, a finding consistent with the established role of oxidative metabolism in generating a metabolically hostile microenvironment that exhausts infiltrating T cells. The triacylglycerol-rich MPC1 microenvironment provides an abundant lipid substrate pool that MC phospholipases can access to replenish arachidonic acid stores, thereby sustaining eicosanoid output beyond what could be achieved through *de novo* synthesis alone in MCs. Conversely, in the acylcarnitine-enriched MPC3 context, elevated β-oxidation flux within MCs may enhance their production of reactive oxygen species and support the transcriptional activity of HIF-1α, further shifting MC mediator output toward a hypoxia-adapted, immunosuppressive secretory profile. These metabolic subtype-specific interactions between MCs and the broader ESCC metabolic landscape have not been experimentally characterized and represent a compelling translational priority.

The single-cell transcriptomic atlas of ESCC reported provides an additional and mechanistically complementary perspective on how the ESCC metabolic ecosystem shapes immune cell function at the resolution of individual cell types (Zhang et al., 2021). Single-cell transcriptomic profiling of ESCC identifies 42 cell types and complex communication networks between malignant and stromal compartments. Although MCs were not a primary cluster, these interaction networks clarify their metabolic behavior within the tumor microenvironment. Active signaling between fibroblasts and immune cells involving lipid transport suggests co-regulated metabolic programs. Within this framework, MCs likely engage in bidirectional crosstalk with neighboring populations. MC derived PGE2 and LTB4 reprogram macrophages toward an M2 phenotype, while fibroblast secreted CXCL12 and TGF-beta sustain MCs FASN expression and lipid mediator synthesis (Pandey et al., 2024; Moore and Pidgeon, 2017; Xue et al., 2020). The single-cell atlas framework thus reveals that the metabolomic changes in ESCC are not cell-autonomous events but emerge from coordinated, spatially organized intercellular metabolic exchanges in which MCs are likely active participants.

Collective evidence from bulk lipidomic profiling, metabolic subtype stratification, and single-cell ecosystem architecture supports a unified model of the ESCC microenvironment (Zhang D. et al., 2024; Wang et al., 2024c). This environment is organized into specialized niches that differentially regulate MC activation states and their immunosuppressive output. In the lipid-dominant MPC1 niche, MC eicosanoid secretion is amplified by an abundance of arachidonic acid precursors. Conversely, in the energy-dominant MPC3 niche, MC function is shaped by lactate-driven acidification and mitochondrial reprogramming which favor a more profoundly immunosuppressive and therapy-resistant phenotype.

This metabolic niche-dependent model provides a mechanistically grounded framework for future studies to explicitly account for MC contributions to the tumor lipid landscape. Addressing this scientific gap is essential for the rational development of strategies that co-target metabolism and the immune system. These findings indicate that metabolomic and lipidomic reprogramming in ESCC actively instructs MC functional polarization, rather than passively reflecting tumor metabolism.

### 3.5 Integration of catabolic and anabolic pathways in lipid reprogramming

In addition to enhanced lipid biosynthesis, ESCC metabolic reprogramming is sustained by coordinated integration of catabolic and upstream metabolic pathways. Fatty acid β-oxidation represents a critical mechanism through which tumor cells utilize lipids as an energy source. Within mitochondria, β-oxidation generates acetyl-CoA and reducing equivalents that fuel the tricarboxylic acid (TCA) cycle and oxidative phosphorylation, thereby supporting tumor survival under metabolic stress. In parallel, peroxisomal β-oxidation contributes to the processing of very-long-chain fatty acids and maintains lipid homeostasis, highlighting a complementary role between these organelles in sustaining tumor metabolic flexibility (Carracedo et al., 2013).

Under homeostatic conditions, peroxisomal β-oxidation in MCs operates at a basal level sufficient to clear very-long-chain fatty acids (VLCFAs) with a carbon chain length of 22 or more and maintain membrane lipid composition, thereby supporting MC quiescence and tissue surveillance without triggering excessive eicosanoid production (Wanders and Waterham, 2006; Islinger et al., 2018). In this resting state, the acetyl-CoA generated by peroxisomal oxidation is largely directed toward housekeeping lipid turnover rather than inflammatory mediator synthesis, and hydrogen peroxide byproducts are efficiently neutralized by peroxisomal catalase, limiting NF-κB activation (Schrader and Fahimi, 2006). However, this equilibrium is fundamentally disrupted. Chronic exposure to tumor-derived lipid ligands, hypoxic signaling, and persistent KIT or IgE receptor engagement drives a marked upregulation of peroxisomal β-oxidation activity in tumor-infiltrating MCs, generating excess acetyl-CoA that is preferentially channeled into arachidonic acid biosynthesis and subsequently into PGE2 and LTB4 production in ESCC (Wanders and Waterham, 2006; Islinger et al., 2018). Concurrently, the increased peroxisomal H_2_O_2_ load in the ESCC microenvironment saturates catalase-mediated neutralization, allowing oxidative spillover that activates NF-κB and further amplifies MC pro-tumorigenic transcriptional programs (Schrader and Fahimi, 2006). The transition from homeostatic lipid clearance to tumor-promoting eicosanoid amplification thus represents a critical metabolic reprogramming event in ESCC-infiltrating MCs that represents a mechanistically testable axis that may serve as a potential therapeutic target in ESCC.

Beyond lipid degradation, the citrate shuttle provides a direct biochemical link between central carbon metabolism and lipid synthesis. Citrate exported from the mitochondria is converted into cytosolic acetyl-CoA by ATP citrate lyase, supplying the essential substrate for fatty acid and cholesterol biosynthesis. This process functionally connects glycolysis-derived carbon flux to lipid anabolism, thereby reinforcing the metabolic plasticity required for rapid tumor growth (Pavlova and Thompson, 2016).

Under homeostatic conditions, resting MCs exhibit stringent regulation of mitochondrial citrate export via SLC25A1. This flux is maintained at a threshold sufficient for baseline phospholipid turnover, yet insufficient to drive significant *de novo* lipogenesis or eicosanoid synthesis (Zaidi et al., 2012). Acetyl-CoA by ATP citrate lyase activity remains low, and FASN expression is modest, reflecting the metabolic restraint appropriate for tissue-resident sentinel function. In ESCC-infiltrating MCs, this restraint is lost. Hypoxia-driven HIF-1α activation and sustained glycolytic flux increase the availability of citrate for mitochondrial export, while acetyl-CoA by ATP citrate lyase and FASN are coordinately upregulated, channeling cytosolic acetyl-CoA into expanded phospholipid pools and continuous arachidonic acid esterification (Mendoza et al., 2021; Zaidi et al., 2012). This shift directly sustains the high-output secretion of PGE2, LTB4, and PAF into the ESCC microenvironment, establishing a metabolically fueled immunosuppressive loop that is absent under normal mucosal physiology. Consequently, the homeostasis-to-ESCC transition in citrate shuttle activity represents a qualitative reprogramming of MC function from homeostatic maintenance to active immunometabolic suppression. Collectively, these findings position the citrate shuttle as a central metabolic hub linking MC metabolic adaptation to sustained immunosuppressive signaling in ESCC.

Glycolysis and amino acid metabolism further contribute to lipid metabolic reprogramming by supplying key intermediates to the TCA cycle. Enhanced glycolytic flux generates pyruvate, which is converted into acetyl-CoA, while glutamine metabolism provides α-ketoglutarate to sustain TCA cycle activity. These pathways collectively ensure a continuous supply of biosynthetic precursors for lipid production while simultaneously supporting redox balance and energy generation (Pavlova and Thompson, 2016).

The functional integration of catabolic and anabolic pathways in ESCC is further illuminated by recent metabolomic and lipidomic profiling studies. Research employing unsupervised metabolic pathway clustering has stratified ESCC tumors into three distinct metabolic subtypes. These categories comprise the lipid dominant MPC1, the amino acid dominant MPC2, and the energy metabolism dominant MPC3 (Wang et al., 2024c). The MPC1 subtype was characterized by pronounced accumulation of triacylglycerols and complex lipid species consistent with enhanced lipid anabolic flux, whereas MPC3 exhibited heightened glycolysis and carnitine accumulation, reflecting preferential reliance on fatty acid oxidation and mitochondrial energy production. Notably, MPC3 demonstrated the poorest clinical response to PD-L1 immunotherapy, suggesting that a catabolic metabolic state dominated by carnitine-mediated lipid shuttling and oxidative phosphorylation is mechanistically coupled to immune resistance in ESCC (Wang et al., 2024c). The lipid-enriched microenvironment of MPC1 tumors is expected to expand the substrate pool available for MC membrane phospholipid synthesis and arachidonic acid esterification, thereby augmenting eicosanoid secretory capacity beyond what would occur in normal mucosal tissue. Conversely, the glycolytic and oxidative environment of MPC3 tumors may sustain MC activation through lactate-mediated signaling and promote a shift in MC mediator output toward a more profoundly immunosuppressive profile. These considerations imply that the immunometabolic contribution of MCs in ESCC is not uniform but is instead shaped by the dominant metabolic subtype of the surrounding tumor, a hypothesis that if validated experimentally would support metabolic subtype-stratified targeting of MC pathways as a component of precision immunotherapy in ESCC.

**FIGURE 1:**
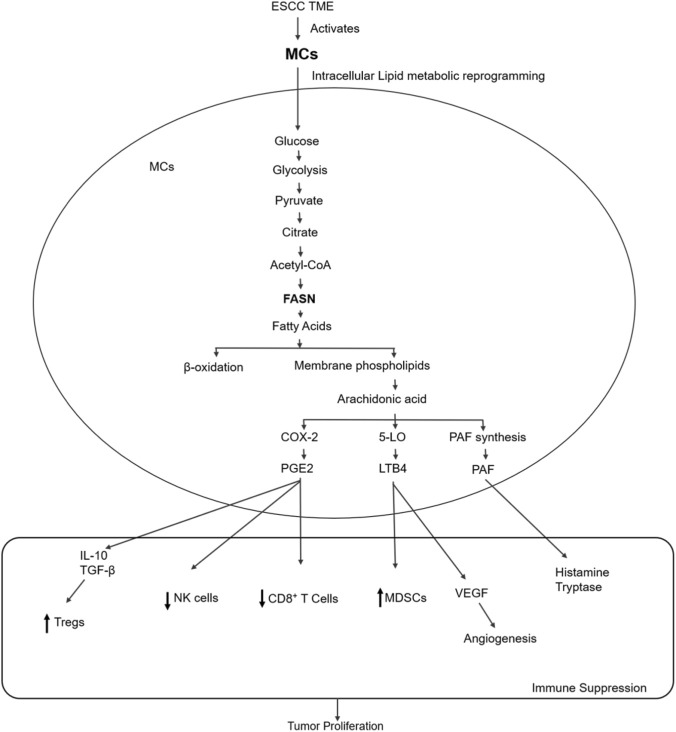
MC-driven lipid metabolic reprogramming coordinates immunosuppressive signaling in ESCC.

These interconnected metabolic pathways exert direct effects on immune cell function within the tumor microenvironment. Elevated fatty acid oxidation is associated with T cell exhaustion and diminished effector activity, whereas disruptions in glucose and amino acid metabolism further constrain T cell activation and persistence. Within this metabolically constrained setting, MCs are positioned in regions characterized by nutrient stress and hypoxia, where they may influence local metabolic flux through the release of lipid mediators and cytokines. Such interactions are likely to reinforce immunosuppressive circuits and sustain immune evasion in ESCC. Taken together, these metabolic adaptations suggest that lipid reprogramming in ESCC extends beyond biosynthesis and energy utilization, ultimately intersecting with immune regulation. In this context, MCs may serve as a critical node linking metabolic rewiring to immunosuppressive signaling (Figure 1).

Within the ESCC TME, MCs undergo lipid metabolic reprogramming characterized by enhanced glycolytic flux, citrate export, and FASN. Fatty acids are incorporated into membrane phospholipids, which serve as a reservoir for arachidonic acid. Upon activation, arachidonic acid is metabolized through COX-2, 5-LO, and PAF related pathways, leading to the production of lipid mediators, including PGE2, LTB4, and PAF. Activated MCs act as a major source of these mediators and release them into the surrounding microenvironment. PGE2 directly suppresses CD8^+^ T cell and NK cell activity, while also promoting the production of IL-10 and TGF-β, thereby supporting Treg expansion. Meanwhile, LTB4 contributes to the accumulation of MDSCs, whereas PAF is linked to VEGF induction and angiogenesis. Additional MCs derived factors, such as histamine and tryptase, further modulate the TME. Together, these coordinated metabolic and signaling pathways establish an immunosuppressive niche that favors tumor progression in ESCC.

## 4 Immune microenvironment remodeling in ESCC

MCs function as strategic environmental sentinels in ESCC, localized within hypoxic and lipid-rich niches to transduce metabolic cues into potent immunomodulatory signals (Zhang S. et al., 2024). Upon activation, MCs release a bioactive secretome composed of prostaglandins, leukotrienes, and PAF, which drives stromal remodeling and disrupts the metabolic fitness and effector function of infiltrating CD8^+^ T cells (Moore and Pidgeon, 2017; Mao et al., 2021). This MC-driven immunometabolic axis facilitates a suppressive architecture that attenuates antigen presentation and enforces immune exclusion (Ribatti, 2024). Consequently, MC activation fundamentally reshapes the local leukocyte landscape, creating conditions permissive to tumor persistence and immune evasion.

### 4.1 Immune-cell composition

MC-enriched tumor regions in ESCC frequently coincide with diminished CD8^+^ T cell density and a concomitant influx of regulatory subsets. Elevated infiltration of Tregs and MDSCs is robustly associated with adverse prognosis in ESCC (Zhang M. et al., 2023). This suppressive expansion is driven largely by MC-derived lipid mediators such as PGE2 and leukotrienes, which facilitate the recruitment and expansion of these suppressive populations. This metabolic-immune signaling axis effectively neutralizes cytotoxic defenses to promote immune escape. Spatial transcriptomic characterizations in other solid malignancies suggest that MCs often cluster with fibroblasts and endothelial cells to form immune-stromal hubs that act as immunologically sequestered compartments. Whether a comparable MC-centered niche exists in ESCC remains a critical knowledge gap requiring high-resolution multi-omic clarification.

### 4.2 Cytokine-chemokine signaling and phenotypic plasticity

Beyond lipid-mediated signaling, MCs release a diverse repertoire of cytokines and chemokines that progressively reshape the ESCC immune ecosystem. MC-derived PGE2 serves as a multi-modal regulator, suppressing dendritic cell differentiation and antigen-presentation while skewing macrophages toward a pro-tumorigenic M2 phenotype (Moore and Pidgeon, 2017). Concurrently, this eicosanoid signaling impairs the cytolytic potency of CD8^+^ T cells and fortifies the Treg compartment to reinforce local immune tolerance (Moore and Pidgeon, 2017). Similarly, leukotriene signaling facilitates the accumulation of MDSCs, contributing to a profoundly suppressive milieu (Moore and Pidgeon, 2017). Emerging evidence indicates that MCs enriched in IL-10 can function as localized cytokine reservoirs with potential systemic implications, including the dampening of immune priming within tumor-draining lymph nodes. Given the infiltrative and stroma-dense architecture of ESCC, such MC-dependent mechanisms are biologically plausible and warrant systematic investigation in clinical cohorts.

### 4.3 Metabolic signals that reprogram immune fate

**FIGURE 2:**
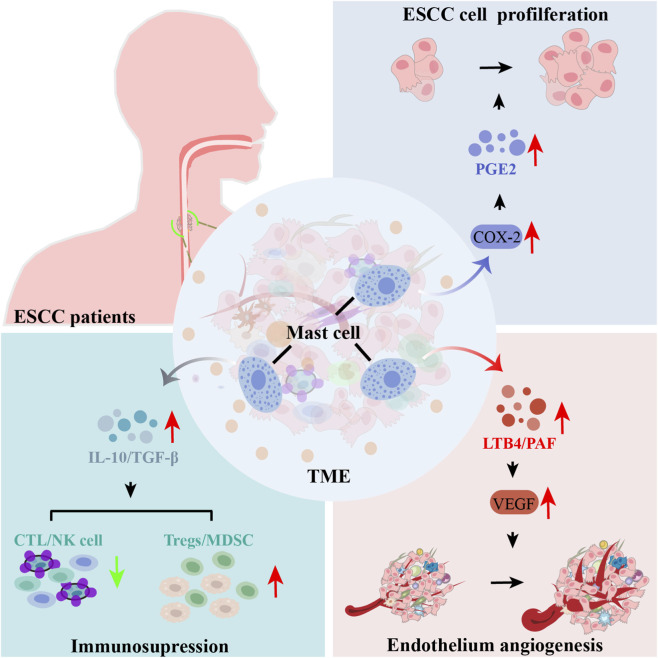
MC associated lipid mediators orchestrate ESCC tumor growth, angiogenesis, and immune suppression within the TME. MCs accumulate within stromal and perivascular niches of the ESCC TME and, upon activation, release a repertoire of lipid mediators including PGE2, LTB4, and PAF, as well as immunoregulatory cytokines such as IL-10 and TGF-β. These mediators support tumor cell proliferation through COX-2 signaling and promote vascular expansion by inducing VEGF expression in endothelial cells. Concurrently, MC-derived lipids and cytokines weaken antitumor immunity by reducing cytotoxic T lymphocyte and natural killer cell activity and by facilitating the recruitment and functional reinforcement of Tregs and MDSCs. Collectively, these MCs driven pathways establish an immune-refractory and metabolically conditioned microenvironment that enables tumor persistence and progression in ESCC.

MC-derived eicosanoids, particularly PGE2, function as primary metabolic ligands within the ESCC microenvironment. Signal transduction through prostaglandin E receptor 1 to prostaglandin E receptor 4 (EP1 to EP4) receptors initiates intracellular cascades such as the phosphoinositide 3-kinase (PI3K)/protein kinase B (AKT) and mitogen-activated protein kinase (MAPK) pathways which accelerate tumor cell proliferation and facilitate migratory phenotypes (Jiang et al., 2004). In ESCC, the persistent upregulation of the COX-2/PGE2 axis remains a robust correlate of heightened tumor invasiveness and adverse clinical outcomes (Figure 2) (Jiang et al., 2004). Complementing the pro-tumorigenic effects of PGE2, MCs mobilize leukotrienes including LTB4 and cysteinyl leukotrienes to promote EMT and facilitate tissue invasion (Liu et al., 2025; Zhang GC. et al., 2023; Moore and Pidgeon, 2017). Furthermore, PAF signaling mediated via the platelet-activating factor receptor (PAFR) drives angiogenesis and biomass expansion (Moore and Pidgeon, 2017). While direct evidence in ESCC is currently emerging, the documented expression of PAFR in esophageal lineages suggests a pathogenic role for PAF in stimulating VEGF production and promoting microvessel formation, consistent with observations in breast and lung malignancies (Bussolati et al., 2000; Chen et al., 2015).

The relationship among PGE2, LTB4, and PAF is not simply additive. These mediators engage in both synergistic and antagonistic interactions that shape the overall immunosuppressive output of the MC secretome. At the level of intracellular signaling, PGE2 and PAF converge on partially overlapping downstream pathways. PGE2 activates adenylyl cyclase and PI3K through EP2 and EP4 receptors, whereas PAF engages PI3K and MAPK signaling via PAFR, thereby promoting tumor cell survival and angiogenic responses (Moore and Pidgeon, 2017; Chen et al., 2015). In addition, PGE2 has been reported to enhance PAFR expression on target cells, which may increase cellular responsiveness to PAF and contribute to a feedforward amplification loop within the TME.

At the level of immune regulation, PGE2 and LTB4 appear to exert complementary rather than redundant functions. PGE2 suppresses dendritic cell maturation and CD8^+^ T cell activity through EP receptor signaling, while LTB4 promotes the recruitment and accumulation of myeloid-derived suppressor cells via BLT1 engagement (Moore and Pidgeon, 2017). Together, these mediators affect distinct nodes of the antitumor immune response, including antigen presentation and cytotoxic effector function. LTB4 and PAF may also cooperate in shaping myeloid cell recruitment. Neutrophils recruited by LTB4 can produce PAF under inflammatory conditions, suggesting the existence of a transcellular amplification loop that extends the initial MC-derived signal within the tumor stroma.

An important antagonistic interaction occurs at the level of arachidonic acid metabolism. COX-2 and 5-lipoxygenase compete for the same substrate pool within MCs. When COX-2 activity is inhibited, arachidonic acid availability may increase for 5-LO mediated metabolism, potentially leading to compensatory increases in leukotriene production. This metabolic shift has been proposed as one factor contributing to the limited efficacy of COX-2 inhibition alone in tumor settings and suggests that combined targeting of COX-2 and leukotriene pathways may be required for more effective disruption of lipid-mediated immunosuppression (Moore and Pidgeon, 2017).

Collectively, these synergistic, antagonistic, and transcellular interactions among PGE2, LTB4, and PAF establish a functionally integrated immunometabolic network that is more resilient to single-mediator intervention than the individual contributions of each molecule alone.

### 4.4 Unresolved clinical immunology and knowledge gaps

Recent findings emphasize that MCs not only secrete soluble lipid mediators but also release exosomes and microvesicles enriched in lipids and lipid-modified proteins (Groot et al., 2016), facilitating intercellular communication and transferring oncogenic signals within the TME. Evidence from other tumor types further supports a functional role for MCs in shaping the immunometabolic landscape. MC infiltration correlates with enhanced fatty acid metabolism and poor survival in pancreatic ductal adenocarcinoma (PDAC) (Chang et al., 2011), and MC-rich niches enriched in IL-10 and lipid factors establish immune-refractory microenvironments that confer resistance to immunotherapy (Ma et al., 2024). Additionally, MCs utilize 5-lipoxygenase (5-LO) to produce LTB4, which promotes immune evasion by recruiting MDSCs in colon cancer (Cheon et al., 2011). Although direct studies are limited, several lines of indirect evidence in ESCC including high COX-2/PGE2 signaling (Jiang et al., 2004), marked immunosuppressive cell burden (Zheng et al., 2020), and dysregulated lipid metabolism (Si et al., 2024), support MC involvement. Immunohistochemical studies have identified MCs in proximity to tumor cells and vasculature in ESCC specimens (Wang et al., 2013).

Despite these mechanistic insights, the therapeutic landscape targeting MC-driven immunometabolism in ESCC remains largely unexploited. COX-2 inhibitors, leukotriene receptor antagonists, and PAFR inhibitors demonstrate antitumor effects in preclinical models (Segura-Villalobos et al., 2022), and their combination with immune checkpoint inhibitors may synergistically reverse lipid-mediated immune suppression (Ping et al., 2025). Future translational priorities include identifying MC-dependent biomarkers predicting immunotherapy response and evaluating whether dual targeting of FASN-driven lipid synthesis and MC lipid signaling can resensitize ESCC to PD-1 blockade.

### 4.5 MC-derived mediators and stromal redundancy

A key mechanistic question is whether targeting MCs alone is sufficient to disrupt the immunosuppressive microenvironment in ESCC, given that mediators such as PGE2, IL-10, and TGF-β are also produced by tumor-associated macrophages, cancer-associated fibroblasts, and Tregs. As summarized in Table 2, although multiple stromal and tumor cell types contribute to these mediators, MCs display distinct kinetic and functional features that differentiate their role within this network. Current evidence suggests that MC-derived mediators may occupy a temporally and functionally privileged position (Zhang D. et al., 2024; Moore and Pidgeon, 2017; Qu et al., 2025). MCs are distinguished by their ability to rapidly release preformed mediators through degranulation, enabling the delivery of lipid mediators and proteases within minutes of activation, whereas macrophage polarization and fibroblast activation depend on transcriptional reprogramming over longer time scales (Mukai et al., 2018; Moore and Pidgeon, 2017). This kinetic property supports a role for MCs as early initiators of immunosuppressive signaling rather than passive participants. In addition, certain mediators show relatively restricted cellular origins within the ESCC microenvironment. PAF production at biologically relevant levels is largely confined to MCs and activated platelets, with platelet-derived PAF being primarily linked to thrombosis rather than immunoregulation (Zhao et al., 2024; Chen et al., 2015). LTB_4_ production through the 5-LO pathway is mainly attributed to MCs and neutrophils, but neutrophil-derived LTB_4_ is highly context-dependent and often occurs following prior immune cell recruitment, a process that may itself be influenced by MC activity (Moore and Pidgeon, 2017; Cheon et al., 2011). Beyond these features, MC-derived signals may also act upstream of other immunosuppressive pathways. PGE2 has been shown to promote M2 macrophage polarization and subsequent IL-10 production, while MC proteases can activate latent TGF-β in the extracellular matrix and facilitate Treg differentiation (Pandey et al., 2024; Xue et al., 2020). Together, these observations suggest that reducing MC mediator output could attenuate both direct and secondary immunosuppressive signals.

**TABLE 2:** Cellular sources of key immunosuppressive mediators in the ESCC TME and the specific contribution of MCs.

Mediator	MC contribution	Other TME sources	MC specific features	References
PGE2	COX-2^+^ lipid-enriched MCs act as early stromal contributors following IgE or KIT activation	Tumor cells, M2 TAMs, CAFs	Rapid COX-2 activation and preformed arachidonic acid pools enable faster PGE2 release compared with *de novo* synthesis in TAMs	Jiang et al. (2004), Wilson and DuBois (2022), Moore and Pidgeon (2017), Qu et al. (2025)
LTB4	5-LO^+^ MCs represent a major cellular source in tumor tissues	Neutrophils, macrophage subsets	Co-secretion with PAF is characteristic of MCs, whereas neutrophil-derived LTB_4_ is context-dependent and requires prior recruitment	Moore and Pidgeon (2017), Cheon et al. (2011)
PAF	MCs are among the few cell types with constitutive PAF synthesis capacity	Platelets, endothelial cells	Platelet-derived PAF is primarily associated with thrombosis, while MC-derived PAF is linked to immunomodulatory signaling; PAFR is expressed in ESCC epithelium	Zhao et al. (2024), Chen et al. (2015)
IL-10	Immunosuppressive MC subsets expressing IL10 and TGFB1 may function as localized cytokine reservoirs	M2 TAMs, Tregs, MDSCs	MC-derived IL-10 may precede and facilitate Treg accumulation, while Treg-derived IL-10 likely represents a downstream amplification step	Zhang et al. (2024a), Xue et al. (2020)
TGF-β	MCs expressing TGFB1 contribute to sustained TGF-β production	CAFs, Tregs, tumor cells	MC-derived proteases can activate latent TGF-β in the extracellular matrix, providing a mechanism independent of transcriptional secretion	Xue et al. (2020), Li et al. (2022)
VEGF	Hypoxia-associated MCs with HIF-1α activation contribute to early VEGF release	Tumor cells, CAFs, TAMs	VEGF release from MCs is coupled to degranulation and occurs rapidly, whereas other sources rely on transcriptional induction	Segura-Villalobos et al. (2022), Jones et al. (2019)

At the same time, partial compensation by other cellular sources cannot be excluded. Tumor cells can sustain COX-2-driven PGE2 production, and established fibroblast populations may maintain TGF-β signaling independently of MC input (Jiang et al., 2004; Wilson and DuBois, 2022). These observations indicate that targeting MCs alone may not be sufficient in all settings. Instead, MC directed strategies are more likely to be effective when applied early or combined with approaches that target tumor-intrinsic or stromal compensatory pathways.

## 5 MCs shape tumor plasticity in ESCC

MCs function as passive stromal residents or active drivers of immune exclusion in ESCC requires a detailed synthesis of their spatial distribution and transcriptional identity.

### 5.1 Spatial localization and single-cell atlas characterization

MCs originate from hematopoietic progenitors and complete terminal maturation under specific stromal cues within peripheral tissues. The scRNA-seq analyses in ESCC have identified MC clusters characterized by the expression of IL-10, TGFB1, and Prostaglandin-endoperoxide synthase 2 (PTGS2), which directly implicate these cells in immunoregulatory and lipid-synthetic programs (Zhang D. et al., 2024). Spatial transcriptomic data further demonstrate MC enrichment within perivascular and hypoxic domains where nutrient depletion and angiogenic signaling predominate. These MC-dense niches frequently coincide with exhausted CD8^+^ T cells and Tregs, suggesting a functional role in local immune dysfunction. In other malignancies, the physical proximity of MCs to fibroblasts facilitates the formation of stromal-immune hubs that act as immune-privileged compartments or tertiary tolerance zones (Guo W. et al., 2025). The existence of similar architectural features in ESCC remains a critical knowledge gap that necessitates further multi-omic validation.

### 5.2 Metabolic rewiring driven by MC mediators

While IL-10, TGF-β, histamine, and VEGF contribute to the baseline immunologic tone, lipid-derived mediators appear particularly influential in the context of ESCC. PGE2 suppresses the maturation of antigen-presenting cells and disrupts T cell receptor (TCR)-mediated signaling in CD8^+^ T cells (Komi and Redegeld, 2020; Portales-Cervantes et al., 2019; Sreeramkumar et al., 2012; Shi Z. et al., 2019; Cui et al., 2017). Furthermore, MC-derived PGE2 has been shown to accelerate fatty-acid biosynthesis and expand lipid reservoirs, which provide essential bioenergetic support for tumor cells under metabolic stress (Qu et al., 2025). Other mediators such as LTB4 and cysteinyl leukotrienes facilitate EMT and tissue invasion (Moore and Pidgeon, 2017; Zhang M. et al., 2023). PAF similarly promotes biomass expansion and angiogenesis, and its cognate receptor PAFR is detectable in the ESCC epithelium (Zhao D. et al., 2023). This observation supports a model in which MC-derived lipids establish a metabolically conditioned barrier that restricts effector cell function. Concurrently, MC-driven stromal remodeling promotes desmoplastic reorganization by fibroblasts, physically impeding lymphocyte infiltration.

### 5.3 Modulation of immune lineage fate

The secretion of IL-10 and TGF-β by MCs effectively impairs dendritic cell function and suppresses the proliferation of cytotoxic T cells while promoting the induction of Tregs (Xue et al., 2020; Li et al., 2022; Mirlekar, 2022; Veen et al., 2021). Histamine biases Th1/Th2 differentiation toward tumor-permissive phenotypes (Portales-Cervantes et al., 2019; Shi Z. et al., 2019). Recent studies demonstrate that MCs can function as immune gatekeepers, influencing which immune cell lineages receive metabolic fuel and which undergo exhaustion. The scRNA-seq observations suggest MCs may also express checkpoint ligands including PD-L1, enabling direct suppression of T cell function (Lai et al., 2025), although ESCC-specific validation remains absent and warrants dedicated investigation. Collectively, these findings position MCs as multifunctional regulators of immune lineage fate, capable of simultaneously suppressing cytotoxic responses and reinforcing tolerance through both soluble mediators and direct cell-surface interactions.

### 5.4 The MC immunometabolic axis

The MC-driven immunometabolic axis is better viewed as a layered process that unfolds across different levels of regulation. It does not operate as a simple linear pathway. Instead, it reflects how metabolic adaptation in MCs gradually translates into broader changes within the ESCC microenvironment.

At the cellular level, MCs respond to metabolic stress by adjusting their internal programs. Under hypoxic and nutrient-limited conditions, they increase lipid biosynthesis and glycolytic activity, which supports sustained secretory function. Enzymes such as FASN and ACACA are upregulated in this context, allowing MCs to maintain a high level of mediator production despite environmental constraints (Mendoza et al., 2021; Zhang S. et al., 2024).

This metabolic adaptation is closely linked to what these cells release into the surrounding tissue. MCs continuously produce a mixture of lipid mediators and cytokines, including PGE2, LTB4, PAF, IL-10, and TGF-β, which act on nearby immune and stromal cells. PGE2 has been associated with impaired dendritic cell maturation and a shift in macrophages toward an M2-like phenotype (Pandey et al., 2024). LTB4 contributes to the recruitment and expansion of myeloid-derived suppressor cells (Pascual and Benitah, 2024). At the same time, IL-10 and TGF-β support the accumulation of Tregs and reinforce an immunosuppressive environment (Mukai et al., 2018; Zhang D. et al., 2024). MC-derived proteases, particularly tryptase, further contribute to this process by activating fibroblasts and promoting stromal remodeling, thereby reinforcing both physical and functional barriers within the tumor niche (Oskeritzian, 2012).

When these effects are considered together, a pattern begins to emerge. Many immune and stromal cells participate in immunosuppression, but they tend to act after activation signals are already in place. Tumor-associated macrophages, MDSCs, and Tregs mainly execute suppressive functions once they are recruited or polarized. Cancer-associated fibroblasts reshape the extracellular matrix in response to these signals. MCs appear to occupy an earlier position in this sequence. By linking metabolic activity to mediator release, they can influence several downstream pathways at the same time, rather than acting within a single functional compartment.

Their spatial distribution further supports this idea. MCs are often located near blood vessels and in regions where oxygen tension is low. These are areas where metabolic stress is most pronounced, and where local signals can be rapidly amplified. In such settings, increased production of lipid mediators may serve as a bridge between metabolic imbalance and immune regulation, allowing MCs to shape the surrounding environment in a coordinated way (Segura-Villalobos et al., 2022; Li et al., 2024).

Taken together, these observations suggest that MCs function as upstream coordinators within the ESCC microenvironment. Framing their activity as an immunometabolic axis helps integrate metabolic and immune processes into a single model. It also makes clearer how MCs differ from other cell populations that contribute to tumor progression, addressing a gap in how these interactions are often described in ESCC.

### 5.5 Epigenetic reprogramming mediated by MCs

Beyond metabolic and cytokine-mediated regulation, emerging evidence suggests that MCs may also contribute to tumor progression through epigenetic reprogramming within the ESCC tumor microenvironment. Epigenetic modifications, including DNA methylation, histone modifications, and chromatin remodeling, represent critical mechanisms by which tumor and immune cell phenotypes are dynamically regulated in response to environmental cues (Jones et al., 2019; Veen et al., 2021). The epigenetic regulation governs key functional programs including KIT/CD117 expression, cytokine secretion competency, and eicosanoid biosynthetic capacity. Under homeostatic conditions, the KIT locus in MCs is maintained in a transcriptionally permissive chromatin state, with active histone marks including H3K4me3 and H3K27ac at the promoter region, ensuring constitutive surface expression of CD117 and sensitivity to stem cell factor (SCF)-mediated survival signaling (Helou et al., 2022; Cildir et al., 2017). However, tumor-derived TGF-β and PGE2 can progressively remodel the chromatin architecture at the KIT locus in stromal MCs, shifting the balance toward repressive histone marks including H3K27me3 deposited by EZH2, which attenuates KIT transcription in a subset of MCs and may contribute to the emergence of KIT-low, epigenetically reprogrammed MC populations with altered degranulation thresholds and mediator profiles in the ESCC microenvironment (Jiao et al., 2024; Mendoza et al., 2021). The epigenetic silencing of KIT in tumor-conditioned MCs carries significant clinical relevance. MCs with low KIT expression are predicted to exhibit reduced sensitivity to inhibitors such as imatinib or masitinib. This phenomenon potentially explains why KIT-directed strategies achieve only incomplete MC depletion in certain tumor contexts.

MCs can influence these processes indirectly through the secretion of cytokines such as IL-10 and TGF-β, which have been shown to induce epigenetic alterations in immune cells, leading to sustained immunosuppressive phenotypes (Xue et al., 2020; Li et al., 2022; Veen et al., 2021; Mirlekar, 2022). TGF-β signaling is associated with chromatin remodeling events that promote Treg differentiation and suppress cytotoxic T cell activity, thereby reinforcing immune tolerance (Veen et al., 2021).

Beyond immune cells, TGF-β secreted by tumor infiltrating MCs acts directly on malignant epithelial cells to recruit the transcriptional repressor Smad family member 2/3-Histone deacetylase 1 (SMAD2/3-HDAC1) complex to the promoters of E-cadherin and other epithelial identity genes. This recruitment induces transcriptional silencing through histone deacetylation and promotes the acquisition of a mesenchymal and invasive phenotype (Lamouille et al., 2014; Padua and Massagué, 2009). Simultaneously, MC derived TGF-β activates DNA Methyltransferase 3A (DNMT3A) and DNA Methyltransferase 3B (DNMT3B) in cancer cells to drive *de novo* CpG methylation at the promoters of tumor suppressor genes including runt-related transcription factor 3 (RUNX3), cyclin-dependent kinase inhibitor 2A (CDKN2A), and Ras association domain family member 1A (RASSF1A). These loci are among the most frequently hypermethylated regions in clinical specimens (Kaz and Grady, 2014). This epigenetic relay from MC cytokine secretion to promoter hypermethylation in the malignant epithelium represents a mechanistic route by which MCs imprint durable and heritable transcriptional changes on tumor cells that persist after the initial signaling dissipates.

MC-derived lipid mediators constitute an equally important but underappreciated epigenetic signaling axis acting on ESCC cancer cells. PGE2, the dominant eicosanoid released by PTGS2-high MC subsets, signals through EP2 and EP4 receptors on malignant epithelial cells to elevate intracellular cyclic adenosine monophosphate (cAMP), which activates protein kinase A (PKA) and subsequently phosphorylates the histone acetyltransferase (HAT) CBP/p300 (Jiang et al., 2004; Topper et al., 2020). Phospho-CBP/p300 deposits H3K27ac at the promoters and enhancers of oncogenic targets including myelocytomatosis oncogene (MYC), snail family transcriptional repressor 1 (SNAI1), and vascular endothelial growth factor A (VEGFA), transcriptionally licensing their expression and reinforcing a proliferative, pro-angiogenic, and invasive gene program in ESCC cells (Xu C. et al., 2014). Concurrently, PGE2-PKA signaling suppresses the activity of class I and class II HDACs in cancer cells, particularly HDAC1, HDAC2, and HDAC3, which normally restrain the expression of genes involved in EMT and immune checkpoint ligand display (Topper et al., 2020). The net effect of PGE2-mediated HDAC inhibition in ESCC tumor cells is therefore a permissive epigenetic state characterized by globally elevated histone acetylation at oncogenic loci, increased PD-L1 transcription through HAT-mediated enhancer activation, and reduced expression of antigen presentation machinery through HDAC-dependent silencing of major histocompatibility complex class I (MHC-I) genes (Xu C. et al., 2014). This PGE2-HAT/HDAC axis in cancer cells connects MC lipid mediator output directly to the epigenetic enforcement of immune evasion in the malignant epithelium. This mechanistic link is strongly supported by converging evidence from colorectal, lung, and breast cancer systems although it has not yet been experimentally validated in models specific to esophageal squamous cell carcinoma.

Histamine, another preformed MC mediator released during degranulation, provides a further mechanistic route connecting the MC secretome to histone modification in cancer cells. Histamine signals through H2 receptors (HRH2) expressed on ESCC epithelial cells to activate adenylyl cyclase, elevate cAMP, and drive PKA-mediated phosphorylation of cAMP response element-binding protein (CREB), which in turn recruits CBP/p300 to CREB-responsive gene promoters including those controlling cell survival, proliferation, and angiogenesis (Yog et al., 2010). Meanwhile, histamine has been reported to suppress HDAC activity in target cells through a PKA-dependent mechanism, thereby reinforcing the global histone hyperacetylation state initiated by PGE2, and to modulate ten-eleven translocation (TET) enzyme activity in a manner that may alter 5-hydroxymethylcytosine (5hmC) distribution at gene regulatory regions (Yog et al., 2010). Although direct evidence for this pathway in ESCC is currently absent, histamine receptor HRH2 is expressed in esophageal epithelium, and the downstream convergence of histamine and PGE2 on shared PKA-CBP/p300 signaling suggests that these two MC-derived mediators cooperate to establish a reinforced epigenetic state in ESCC cancer cells that is more durable than either mediator alone could achieve.

MC-derived tryptase adds a further layer of epigenetic regulation through protease-activated receptor 2 (PAR-2) signaling on ESCC stromal fibroblasts. PAR-2 activation by tryptase drives fibroblast-to-myofibroblast trans-differentiation, a process that involves EZH2-mediated H3K27me3 deposition at epithelial gene loci and global hypomethylation of pro-inflammatory and matrix-remodeling gene promoters in the activated fibroblast (Bagher et al., 2018). These epigenetically reprogrammed cancer-associated fibroblasts then secrete a secondary wave of TGF-β, hepatocyte growth factor (HGF), and stromal-derived factor-1 (SDF-1/CXCL12), which act on adjacent ESCC malignant cells to sustain the DNMT3A/B-driven promoter hypermethylation of tumor suppressor loci described above. This MC tryptase PAR-2 CAF cancer cell epigenetic relay constitutes a stromal amplification loop in which an initial MC activation event is transduced into durable and multi cell type epigenetic remodeling across the tumor niche. This biological scenario carries significant therapeutic implications because it suggests that epigenetic changes in malignant cells cannot be fully reversed without simultaneously targeting the upstream signals derived from MCs that maintain them.

Recent studies in other tumor types indicate that MC-associated signaling can reshape the epigenetic landscape of both tumor and stromal cells, promoting tumor plasticity and therapeutic resistance (Mendoza et al., 2021; Ma et al., 2024; Gao et al., 2025). Although direct evidence in ESCC remains limited, the convergence of metabolic, immune, and epigenetic regulation across the mechanistic axes described above suggests that MCs function as upstream coordinators of multi-layered tumor adaptation, imprinting changes on cancer cells, fibroblasts, and immune populations simultaneously through a combination of cytokine, lipid mediator, protease, and metabolite-mediated epigenetic signals. The central implication of this integrated model is that the epigenetic footprint of MC activity in esophageal squamous cell carcinoma is not confined to any single cell type or regulatory layer. Instead, this influence propagates across the tumor ecosystem in a spatially organized and temporally persistent manner. This conclusion fundamentally reframes MC targeted therapy as an epigenetic intervention rather than merely an immunological one.

Therefore, targeting epigenetic regulators in combination with MC-mediated signaling pathways may provide a novel strategy to overcome immune resistance. Specifically, the combination of HDAC inhibitors such as vorinostat or entinostat with MC-targeted agents including KIT inhibitors or COX-2 inhibitors may disrupt the PGE2-HDAC suppression axis in ESCC cancer cells while simultaneously reducing the MC-derived upstream signals that maintain it, providing a rationale for a mechanistically integrated epigenetic-immunometabolic combination strategy in ESCC (Falkenberg and Johnstone, 2014; Wang et al., 2025). The integration of epigenetic therapy with metabolic and immunological interventions may therefore represent a promising direction for future ESCC treatment paradigms (Jones et al., 2019; Topper et al., 2020). To further integrate these findings with MC heterogeneity, representative MC subpopulations and their associated epigenetic and functional characteristics are summarized in Table 3.

**TABLE 3:** MC subpopulations and their phenotypic and functional characteristics in the TME.

MC subtype	Identification features	Representative markers	Key mediators	Functional role in TME	References
MCT (mucosal type)	Tryptase^+^, chymase^−^, enriched in epithelial compartments	KIT, FCER1A, CD63	Tryptase, histamine, LTC4, IL-5, IL-6	Epithelial barrier remodeling, Th2-skewed immune responses, eosinophil recruitment	Lichterman and Reddy (2021), Krystel-Whittemore et al. (2015), Mukai et al. (2018)
MCTC (connective tissue-type)	Tryptase^+^, chymase^+^, enriched in stromal and submucosal regions	KIT, FCER1A, CD88	Tryptase, chymase, CPA3, PGD2	ECM remodeling, angiogenesis, fibroblast activation	Lichterman and Reddy (2021), Mukai et al. (2018), Oskeritzian (2012)
Lipid-enriched MC	High PTGS2 and ALOX5 expression, identified by scRNA-seq	KIT, FCER1A, PTGS2	PGE2, LTB4, LTC4, PAF	Major source of immunosuppressive eicosanoids, promotes tumor lipogenesis, suppresses CD8^+^ T cells	Pandey et al. (2024), Bayerl et al. (2023), Pascual and Benitah (2024), Zhang et al. (2024a)
Hypoxia-associated MC	Enriched in hypoxic and perivascular niches, HIF-1α activation	KIT, FCER1A, HIF1A, CXCR4	VEGF, IL-8, MMP-9	Promotes angiogenesis, recruits suppressive myeloid cells, supports stromal expansion	Segura-Villalobos et al. (2022), Mukai et al. (2018), Li et al. (2024)
Immunosuppressive MC	High IL10 and TGFB1 expression, tumor-infiltrating	KIT, FCER1A, CD274	IL-10, TGF-β, IL-6	Induces Treg differentiation, inhibits CD8^+^ T cells, promotes immune tolerance	Mukai et al. (2018), Zhang et al. (2024a), Mirlekar (2022), Lai et al. (2022)
Pro-inflammatory MC	IL-17^+^ or TNFα-high, associated with favorable ESCC outcomes	KIT, FCER1A, IL17, TNF	IL-17, TNF-α, IFN-γ	Enhances antitumor immunity, recruits cytotoxic lymphocytes	Wang et al. (2013)
Epigenetically reprogrammed MC	Induced by TGF-β and PGE2 signaling	KIT, FCER1A, EZH2, DNMT3A	IL-10, TGF-β, epigenetic regulators	Maintains long-term immunosuppressive states, linked to therapy resistance	Jiao et al. (2024), Mendoza et al. (2021)

## 6 MC-driven immunometabolism as a therapeutic entry point in ESCC

**FIGURE 3:**
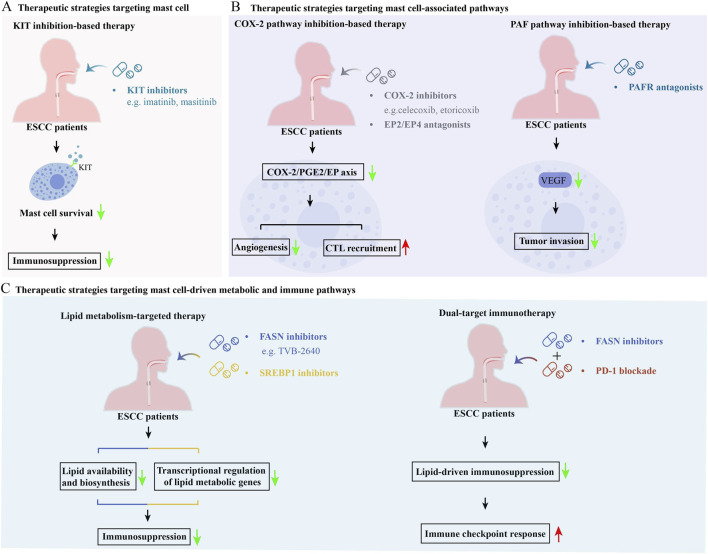
Therapeutic strategies targeting MCs and MC-driven immunometabolism in ESCC. **(A)** Therapies directly targeting MCs. Pharmacologic inhibition of KIT, essential for MC survival and maturation, reduces TME MCs, attenuating MC-dependent immunosuppression. **(B)** Strategies interrupting MC associated signaling pathways. COX-2 pathway suppression decreases PGE2 and lowers angiogenic signaling, improving cytotoxic T lymphocyte infiltration. PAFR antagonists block PAF-mediated tumor invasion and vascular remodeling. **(C)** Approaches targeting MC linked metabolic and immune programs. Inhibiting FASN and SREBP1 decreases lipid biosynthesis and transcriptional activation to reverse lipid conditioned immune suppression within the TME. Dual modality immunotherapy combining metabolic intervention with PD-1 blockade improves immune checkpoint responsiveness and enhances ESCC tumor immunogenicity.

MCs function as upstream coordinators of metabolic fuel availability and immune tone in ESCC, positioning them as a viable therapeutic target. Effective intervention requires a multi-tiered strategy incorporating direct MC depletion, metabolic disruption, and immunomodulatory restoration, as summarized in Figure 3.

### 6.1 Direct targeting of MCs

Current pharmacological strategies primarily utilize the inhibition of the receptor tyrosine kinase KIT to disrupt MC development and survival (Zhang S. et al., 2024). KIT inhibitors such as imatinib and masitinib have demonstrated clinical efficacy in reducing MC density and limiting tumor progression across diverse solid malignancies (Lichterman and Reddy, 2021). Recent pilot characterizations in ESCC indicate that KIT-positive stromal MCs correlate with deeper tissue invasion, suggesting that these cells constitute a distinct pro-tumorigenic subset that warrants stratified depletion rather than non-specific global ablation (Yang et al., 2024). The therapeutic feasibility of this approach depends on whether KIT-positive MCs in ESCC are intrinsically pathogenic or functionally reprogrammed by the TME. Clarifying this distinction through spatial omics-guided patient selection will be essential for the precision targeting of MC populations (Zhang S. et al., 2024).

The need for selective rather than global targeting of MCs reflects their dual roles in physiology and tumor biology. In normal tissues, MCs support host defense, maintain mucosal integrity, and regulate wound healing (Krystel-Whittemore et al., 2015; Oskeritzian, 2012; Ud-Din et al., 2020). Systemic depletion through KIT inhibition may therefore impair these functions, particularly in ESCC patients who are already immunocompromised (Lichterman and Reddy, 2021; Qu et al., 2025). A similar limitation applies to global inhibition of lipid metabolic pathways. COX-2 and FASN are required for physiological signaling in the gastrointestinal, vascular, and renal systems. Clinical use of COX-2 inhibitors has demonstrated gastrointestinal and cardiovascular toxicity, highlighting the risks of non-selective pathway suppression (Wilson and DuBois, 2022; Santiso et al., 2024; da Silva Junior et al., 2018). These constraints make indiscriminate targeting of MCs or eicosanoid signaling unlikely to be clinically sustainable.

Selective targeting strategies are therefore required. First, transcriptional heterogeneity provides a basis for subset-specific intervention. Lipid-enriched MCs with high PTGS2 and ALOX5 expression and immunosuppressive subsets expressing IL10 and TGFB1 are linked to tumor progression, whereas IL-17^+^ subsets may support antitumor immunity (Zhang D. et al., 2024; Guo et al., 2018; Wang et al., 2013). Spatial profiling could enable patient stratification based on dominant MC populations (Chang et al., 2025; Li et al., 2022; Jones et al., 2019). Second, pathway-level targeting offers greater precision. Inhibition of EP4 can disrupt PGE2-mediated immune suppression while preserving broader prostaglandin function. Similarly, 5-LO inhibition may reduce MDSC recruitment without broadly suppressing leukotriene biology (Cuenca-Escalona et al., 2024; Santiso et al., 2024; Nie et al., 2023). Third, local delivery strategies may limit systemic toxicity. Intratumoral or locoregional administration of KIT inhibitors could achieve effective MC depletion within the tumor while minimizing off-target effects (Zhang S. et al., 2024).

Together, these approaches support a shift toward selective modulation of MC function to disrupt tumor-promoting pathways while preserving physiological roles.

### 6.2 Pathway-level modulation of MC mediators

The pharmacological inhibition of MC mediator pathways represents a promising adjunctive therapeutic strategy in ESCC. COX-2 inhibitors such as celecoxib and etoricoxib suppress PGE2 production and attenuate angiogenesis while reversing local immunosuppression in various preclinical models (Leahy et al., 2002; Xu L. et al., 2014). Given that COX-2 overexpression is a robust correlate of adverse prognosis and therapeutic resistance in ESCC, its inhibition constitutes a rational adjunct to existing treatment modalities (Huang et al., 2008). Furthermore, specific antagonists targeting the EP2 and EP4 prostaglandin receptors are under active development and have demonstrated potential in enhancing antitumor immune responses (Francica et al., 2023).

PAFR antagonists represent another emerging class of agents, as studies in lung and breast cancer models have revealed that PAFR blockade effectively attenuates tumor progression (da Silva Junior et al., 2018). Given that MC-derived PGE2 and PAF act as upstream metabolic and immune drivers, inhibiting these pathways may simultaneously interrupt multiple facets of ESCC progression rather than solely limiting angiogenesis (Qu et al., 2025). However, a substantial knowledge gap persists regarding optimal dosing strategies and toxicity profiles for these agents in the ESCC context, and the absence of disease-specific clinical trials underscores the need for translational investigation aimed at identifying predictive biomarkers and establishing safe therapeutic windows (Qu et al., 2025).

The lack of ESCC-specific clinical trials targeting MC related pathways reflect several overlapping challenges. First, unlike gastrointestinal stromal tumors and systemic mastocytosis, activating KIT mutations are uncommon in ESCC. This limits the applicability of KIT-directed therapies in an unselected patient population (Lichterman and Reddy, 2021; Mukai et al., 2018).

Second, the evaluation of MC infiltration remains inconsistent across studies. Variability in antibody selection, sampling regions, and scoring criteria has prevented the establishment of standardized thresholds for patient stratification (Krystel-Whittemore et al., 2015; Oskeritzian, 2012; Wang et al., 2013). In addition, agents such as COX-2 inhibitors have mainly been studied as general anti-inflammatory therapies in ESCC. Their specific impact on MC-driven immunometabolic remodeling is still not well defined (Pandey et al., 2024; Bayerl et al., 2023; Pascual and Benitah, 2024; Mendoza et al., 2021). To address these gaps, a stepwise strategy may be more practical. In the near term, retrospective quantification of MC density in existing immunotherapy cohorts could help determine whether MC infiltration correlates with response to PD-1 blockade (Zhang D. et al., 2024; Wang et al., 2013). Patient-derived organoid and co-culture systems may offer a feasible approach to generate ESCC-specific mechanistic data on MC mediated interactions (Mendoza et al., 2021; Mirlekar, 2022). Those biomarker-guided early-phase trials, for example, combining COX-2 inhibition with PD-1 blockade in patients with high MC infiltration, could provide initial clinical validation of this concept in the longer term (Pandey et al., 2024; Bayerl et al., 2023; Pascual and Benitah, 2024; Lai et al., 2022).

### 6.3 Metabolic targeting and lipid reprogramming

Pharmacological inhibitors of FASN such as TVB-2640 exert potent antitumor effects by restricting the lipid availability essential for membrane biogenesis and oncogenic signaling (Falchook et al., 2021). Beyond direct growth impairment, FASN inhibition sensitizes tumors to immune checkpoint blockade by abrogating the immunosuppressive effects driven by aberrant lipid accumulation (Huang J. et al., 2024). The SREBP1 axis represents another viable therapeutic node, as this transcriptional hub governs both fatty acid and cholesterol biosynthesis and is consistently upregulated in lipid-dependent malignancies (Wang et al., 2020; Ma et al., 2022).

Metabolic interventions directly target the MC-supported lipid microenvironment, which is profoundly enriched in ESCC and functions as a metabolic bottleneck governing tumor plasticity under conditions of metabolic stress (Wang et al., 2024a). However, lipid metabolic inhibition in isolation seldom yields durable clinical responses. This lack of sustained efficacy suggests that the metabolic and immunologic signaling programs within MCs are mutually reinforcing nodes rather than independent therapeutic checkpoints (Lai et al., 2025), and that effective intervention will likely require the simultaneous disruption of both metabolic flux and immunosuppressive signaling within the MC compartment. Key pharmacologic strategies targeting the MC immunometabolic axis in ESCC are summarized in Table 4, including representative agents, mechanistic rationale, and ESCC specific evidence to support therapeutic prioritization.

**TABLE 4:** Therapeutic strategies targeting MC-driven immunometabolism in ESCC.

Therapeutic strategy	Representative agents	Mechanistic rationale	ESCC relevant evidence	Model of study	Posology	Side effects	References
FASN inhibition	TVB-2640	Attenuates de novo fatty acid synthesis to restore antigen presentation and sensitize tumors to PD-1 blockade	Established ESCC metabolic rewiring with checkpoint synergy demonstrated in lipid dependent malignancies	Phase I clinical trials, preclinical solid tumor models	Oral, ∼100 mg/day (clinical trial settings)	Fatigue, gastrointestinal symptoms, reversible liver enzyme elevation	Wang et al. (2024a), Falchook et al. (2021)
SREBP1 targeting	SREBP1 inhibitors/siRNA	Disrupts transcriptional lipogenic reprogramming to impair biomass accumulation	SREBF1 serves as a primary driver of ESCC aggressiveness and clinical recurrence	In vitro ESCC cell lines, xenograft mouse models	Experimental dosing (siRNA/small molecules)	Limited data, potential metabolic disturbances	Li et al. (2021)
KIT directed MC targeting	Imatinib, Masitinib	Reduces MC survival and activation by inhibiting receptor tyrosine kinase signaling	KIT-positive MC infiltration is associated with advanced invasion patterns in ESCC	Preclinical tumor models, early clinical observations	Oral, standard dosing (imatinib 400 mg/day)	Edema, nausea, hematologic toxicity	Moreira et al. (2018), Zhang et al. (2024b)
COX-2 and EP blockade	Celecoxib, Etoricoxib/EP2-EP4 antagonists	Suppresses PGE2 production to establish an immune permissive niche conducive to PD-1 response	COX-2 expression is prognostic in ESCC and EP signaling suppresses CD8+ T cell infiltration	Preclinical ESCC models, clinical use in inflammation or cancer	Oral, celecoxib 200–400 mg/day	Cardiovascular risk, gastrointestinal toxicity	Jiang et al. (2004), Cuenca-Escalona et al. (2024), Huang et al. (2008)
PAF/PAFR inhibition	Ginkgolide B/PAFR antagonists	Abrogates MC PAF induced angiogenesis and tissue invasion	PAFR signaling is known to promote malignancy and vascular remodeling in esophageal cancer	Preclinical models including lung, breast, esophageal cancer	Experimental dosing	Limited data, possible bleeding risk (platelet-related)	Zhao et al. (2023b), Chen et al. (2015)
Dual metabolic immune integration	TVB-2640 and PD-1 inhibitors	Restores MHC-I expression and reduces lipid accumulation to resensitize tumors to checkpoint blockade	Rationalized by ESCC lipid immune rewiring though direct ESCC clinical trials are currently absent	Preclinical combination studies, early clinical exploration	Combination therapy (dose varies by protocol)	Combined toxicity (immune-related plus metabolic)	Huang et al. (2024a), Yao et al. (2024)

### 6.4 Combined immunotherapy strategies

A dual-modality approach integrating lipid metabolism blockade with immune checkpoint inhibitors represents a critical strategy for ESCC due to the role of lipid-rich tumor niches in fostering immune-silent states that diminish PD-1 sensitivity (Yao et al., 2024). Combining the FASN inhibitor TVB-2640 with anti-PD-1 agents resensitizes tumors to checkpoint blockade by reversing lipid-driven immunosuppression (Guo Z-X. et al., 2025; Huang J. et al., 2024). Furthermore, the strategic targeting of MCs may augment the efficacy of checkpoint therapies by abrogating MC mediated T cell suppression, NK cell inhibition, and Treg expansion (Wang et al., 2025). Such combinatorial approaches are designed to convert immune-cold ESCC lesions into immune-permissive environments characterized by enhanced leukocyte infiltration. Prospective clinical trials are now required to establish whether parameters such as stromal MC density, COX-2/IL-10 molecular signatures, or lipid droplet accumulation can serve as robust predictive biomarkers for patient stratification, thereby enabling rational patient stratification and optimizing the clinical deployment of combined metabolic and immune-based therapies in ESCC (Ren et al., 2025).

Translating combined metabolic and immune targeting into clinical practice requires addressing several unresolved challenges. The optimal sequencing of metabolic and immune interventions remains unclear. Concurrent administration of metabolic inhibitors with PD-1 blockade may increase the risk of overlapping toxicities, whereas a sequential approach may allow metabolic modulation of the TME prior to immune checkpoint inhibition (da Silva Junior et al., 2018; Falchook et al., 2021). In addition, the absence of validated predictive biomarkers complicates patient selection, as unselected ESCC populations are unlikely to uniformly exhibit MC-driven immunometabolic features (Zhang D. et al., 2024; Moore and Pidgeon, 2017). The lack of established pharmacodynamic endpoints, including on-treatment changes in tumor PGE2 levels or immune infiltration, further limits the ability to confirm target engagement in early-stage trials (Qu et al., 2025; Huang J. et al., 2024). To address these challenges, a phased translational framework may provide a rational path forward. In an initial phase, metabolic interventions such as COX-2 inhibitors or fatty acid synthase inhibitors could be administered as a lead-in prior to PD-1 blockade to evaluate safety and biological activity. Pharmacodynamic assessment through paired tumor biopsies, including MC density, tumor PGE2 levels, and CD8^+^ T cell infiltration, may help establish target engagement and early immune remodeling (Jiang et al., 2004; Qu et al., 2025). In a subsequent biomarker-enriched expansion phase, patients with high MC infiltration or elevated COX-2 signaling may be selectively enrolled to assess preliminary efficacy and validate predictive biomarkers, consistent with biomarker-driven trial designs increasingly adopted in immuno-oncology (Wang et al., 2020). Ultimately, randomized studies comparing combination therapy with PD-1 monotherapy in biomarker-defined populations would be required to determine clinical benefit. Integration of circulating biomarkers, including plasma PGE2, urinary leukotriene E_4_, and transcriptomic indices of MC activity, may further support non-invasive monitoring and early detection of resistance (Moore and Pidgeon, 2017; Huang J. et al., 2024). This staged approach allows mechanistic validation at each step and may accelerate the clinical translation of MC targeted immunometabolic strategies in ESCC.

### 6.5 Precision medicine and biomarker development

Advanced analytical platforms including single-cell omics and spatial transcriptomics hold considerable promise for advancing the current understanding of MC functionality within the ESCC TME (Zhang S. et al., 2024). The technologies enable the precise delineation of MC heterogeneity and the identification of distinct pro-tumorigenic versus anti-tumorigenic populations (Zhang et al., 2021). Spatial transcriptomics specifically enables the *in situ* mapping of MC interactions with immune and stromal compartments, which provides critical insights into the architectural organization of immunosuppressive niches (Li et al., 2024). Furthermore, the integration of lipidomic signatures and multi-omic risk scores through computational modeling may offer a clinically scalable method to stratify ESCC patients according to their specific MC immunometabolic burden (Pei et al., 2025). A critical limitation of current approaches is that existing biomarkers are predominantly descriptive rather than actionable. Translating these analytical signatures into actionable therapeutic guidelines requires the systematic integration of biopsy-level histology with circulating lipid metabolite profiling to establish clinically validated decision frameworks (Huang Q. et al., 2024). Translating MC related biomarkers into clinically actionable tools requires addressing two key challenges, the standardization of detection methods and the validation of prognostic thresholds across independent ESCC cohorts. As summarized in Table 5, current evidence is largely derived from small single-institution Immunohistochemistry (IHC) studies and retrospective single-cell sequencing analyses, and the reported findings remain heterogeneous and at times inconsistent across studies (Zhang D. et al., 2024; Wang et al., 2013; Mirlekar, 2022). Notably, different studies rely on distinct marker systems and tissue compartments, which contributes to the lack of consensus criteria for MC density quantification. For example, Table 5 shows that tryptase-based IHC is used to quantify total MC infiltration, whereas CD117 based approaches focus on stromal MC subsets associated with tumor invasion, leading to non-interchangeable results (Moreira et al., 2018; Wang et al., 2013). In addition, variation in tissue sampling regions such as muscularis propria, tumor stroma, and invasive front, together with differences in counting strategies and threshold definitions reported across studies in Table 5, further limits reproducibility and cross-study comparability. To improve consistency, future studies should adopt multi-marker IHC panels that combine anti-tryptase with markers such as PTGS2 or IL-10 to distinguish immunosuppressive subsets and apply these panels to standardized tissue regions based on consensus protocols. Digital pathology-based quantification using validated algorithms may further reduce inter-observer variability and support large-scale cohort analyses.

**TABLE 5:** Clinical and translational studies evaluating MC related biomarkers in ESCC.

Cohort size	Detection method	MC Marker(s)	Tissue region	Key finding	Clinical relevance	References
107	IHC	IL-17, Tryptase	Muscularis propria	High IL-17^+^ MC density correlated with prolonged survival	Favorable prognosis	Wang et al. (2013)
62	IHC	CD117 (KIT)	Tumor stroma	Increased stromal CD117^+^ MCs associated with deeper invasion	Adverse prognosis	Moreira et al. (2018)
Multi-cohort	scRNA-seq	TPSAB1, CPA3, PTGS2, IL10, TGFB1	Tumor-infiltrating regions	Immunosuppressive MC subsets (IL10^+^/TGFB1^+^) enriched in advanced ESCC	Adverse prognosis	Zhang et al. (2024a)
Neoadjuvant cohort	scRNA-seq and IHC	KIT, FCER1A	Tumor core and invasive front	Distinct MC infiltration patterns associated with response to immunochemotherapy	Predictive of treatment response	Xu et al. (2014b)

Beyond histological assessment, circulating and transcriptomic biomarkers provide additional and more standardized measures of MC-related activity. As reflected in Table 5, recent transcriptomic analyses have already identified MC subsets characterized by IL10, TGFB1, and PTGS2 expression that are associated with advanced disease and treatment response (Zhang D. et al., 2024; Xu L. et al., 2014). Plasma PGE2 and urinary leukotriene E_4_ can be quantified using established ELISA and mass spectrometry platforms and may serve as systemic indicators of MC eicosanoid activity in ESCC patients during treatment. At the transcriptomic level, MC-associated gene signatures based on TPSAB1, CPA3, PTGS2, and IL10 can be integrated into existing ESCC multi-omics risk models to generate a tissue-independent index of MC burden (Zhang D. et al., 2024). However, these approaches also lack prospective validation in ESCC-specific clinical settings, and their prognostic or predictive value remains to be defined. Prospective validation in multicenter immunotherapy cohorts, with predefined MC-related thresholds evaluated alongside clinical response, will be essential to move MC biomarkers from descriptive observations toward clinically actionable tools.

## 7 Discussion

MCs have been increasingly recognized as central regulators of tumor progression in ESCC through their dual influence on metabolic remodeling and immune suppression (Shu et al., 2025). Despite this recognition, fundamental biological questions remain unresolved including the characterization of subtype specific MC functions and the phenotypic evolution of these cells across distinct disease stages (Yang et al., 2024). Furthermore, while MC-derived lipid mediators such as prostaglandins and leukotrienes facilitate angiogenesis and immune evasion (Lichterman and Reddy, 2021; Oldford and Marshall, 2015), their temporal production kinetics under hypoxia or nutrient restriction remain poorly defined (Gao et al., 2025). In normal esophageal mucosa and early inflammatory states, MCs function as resident sentinels involved in barrier surveillance and wound repair through mediators such as histamine and tryptase. As the mucosa progresses toward dysplasia and early ESCC, persistent inflammatory stimuli, including microbial products, dietary carcinogens, and hypoxia-related signals, may favor a shift toward sustained eicosanoid secretion. COX-2 overexpression is detectable in premalignant esophageal lesions, suggesting that a PGE2-enriched microenvironment may arise before invasive carcinoma is established (Jiang et al., 2004). Evidence from other gastrointestinal malignancies shows that MC infiltration increases at premalignant or chronically inflamed stages and may contribute to early immune tolerance. As a result, MC-derived eicosanoids and TGF-β may help suppress nascent antitumor immune responses before the full immunosuppressive network is assembled.

In advanced ESCC, single-cell and spatial transcriptomic analyses indicate a further shift toward immunosuppressive and hypoxia-associated MC states, with PTGS2-high and IL10-high clusters enriched in invasive and perivascular regions (Zhang D. et al., 2024; Xu L. et al., 2014). The multi-stage spatial carcinogenesis atlas reported provides a framework to examine MC distribution across disease stages, although MC specific longitudinal analyses are not yet available (Chang et al., 2025). Taken together, current evidence supports the possibility that MCs contribute to immunometabolic dysregulation early in ESCC pathogenesis, but direct stage-resolved validation remains limited. Clarifying whether pathogenic MC subsets emerge progressively during dysplasia or arise only under the metabolic pressure of established tumor stroma will be critical for determining whether MC-targeted strategies are better suited for chemoprevention or for combination therapy in advanced disease.

Pharmacological strategies targeting MC-dependent pathways including COX-2 inhibition and lipid metabolic blockade demonstrate significant potential for restoring antitumor immunity and enhancing checkpoint therapy responses (Cui et al., 2022; Jiang et al., 2004). However, the clinical feasibility of incorporating metabolic immunotherapy combinations in ESCC cohorts remains uncertain, particularly regarding whether systemic MC depletion might compromise host pathogen defense (Qu et al., 2025). Future therapeutic frameworks should therefore prioritize selective modulation of pathogenic MC subsets over broad depletion strategies. Identifying these populations requires spatial transcriptomic profiling focused on PTGS2-high and IL10-high MCs. This strategy aims to dismantle the immunosuppressive environment without compromising the beneficial immune and barrier functions inherent to normal tissue MCs. The viability of such targeted approaches is further supported by the emerging interventions and pathway-specific strategies (Wu et al., 2025).

Advances in single-cell omics have uncovered substantial MC heterogeneity and metabolic specialization (Zhang D. et al., 2024), yet existing datasets lack the longitudinal resolution required to track these changes over time (Yang et al., 2024). Future research must integrate spatial transcriptomics and *in situ* metabolic flux profiling to capture dynamic MC metabolism during therapeutic intervention (Zhao et al., 2024). Additionally, the computational reconstruction of MC stromal interactions may facilitate the development of predictive biomarkers for patient stratification (Yang et al., 2025).

Addressing these unresolved questions will determine whether targeting the MC immunometabolic axis can ultimately facilitate durable precision-based control of this aggressive malignancy.

## Glossary

ESCC: esophageal squamous cell carcinoma
MCs: mast cells
TME: tumor microenvironment
PAF: platelet-activating factor
PAFR: platelet-activating factor receptor
PGE2: prostaglandin E_2_
EP1–EP4: prostaglandin E receptors 1–4
EP4: prostaglandin E receptor 4
**PGD**_**2**_: prostaglandin D_2_
**LTB**_**4**_: leukotriene B_4_
**LTC**_**4**_: leukotriene C_4_
BLT1: leukotriene B_4_ receptor 1
5-LO: 5-lipoxygenase
ALOX5: arachidonate 5-lipoxygenase
LPA: lysophosphatidic acid
S1P: sphingosine-1-phosphate
VEGF: vascular endothelial growth factor
FGF-2: fibroblast growth factor-2
IL-10: interleukin-10
TNF-α: tumor necrosis factor-α
IFN-γ: interferon-γ
TGF-β: transforming growth factor-β
CTLs: cytotoxic T lymphocytes
Tregs: regulatory T cells
MDSCs: myeloid-derived suppressor cells
TAMs: tumor-associated macrophages
CAFs: cancer-associated fibroblasts
NK: natural killer cells
APC: antigen-presenting cells
PD-1: programmed cell death protein 1
PD-L1: programmed death-ligand 1
TCR: T cell receptor
MHC-I: major histocompatibility complex class I
KIT: KIT receptor tyrosine kinase
FASN: fatty acid synthase
ACC: acetyl-CoA carboxylase
ACACA: acetyl-CoA carboxylase alpha
HMGCR: 3-hydroxy-3-methylglutaryl-CoA reductase
SREBP1: sterol regulatory element-binding protein 1
PI3K: phosphoinositide 3-kinase
AKT: protein kinase B
mTOR: mechanistic target of rapamycin
MAPK: mitogen-activated protein kinase
TCA: tricarboxylic acid
EMT: epithelial-mesenchymal transition
scRNA-seq: single-cell RNA sequencing
IHC: immunohistochemistry
TPSAB1: tryptase alpha/beta 1
CPA3: carboxypeptidase A3
FCER1A: Fc epsilon receptor I alpha
MCT: mucosal-type mast cell
MCTC: connective tissue-type mast cell
MMP-9: matrix metalloproteinase-9
HIF1A: hypoxia-inducible factor 1-alpha
PTGS2: prostaglandin-endoperoxide synthase 2
EZH2: enhancer of zeste homolog 2
PDAC: pancreatic ductal adenocarcinoma
VLCFAs: very-long-chain fatty acids
SCF: stem cell factor
PKA: protein kinase A
HAT: histone acetyltransferase
SMAD2/3: Smad family member 2/3
HDAC1: histone deacetylase 1
DNMT3A: DNA Methyltransferase 3A
DNMT3B: DNA Methyltransferase 3B
RUNX3: Runt-related transcription factor 3
CDKN2A: Cyclin-dependent kinase inhibitor 2A
RASSF1A: Ras association domain family member 1A
HRH2: Histamine signals through H2 receptors
cAMP: cyclic adenosine monophosphate
MYC: myelocytomatosis oncogene
SNAI1: snail family transcriptional repressor 1
VEGFA: vascular endothelial growth factor A
HRH2: Histamine signals through H2 receptors
CREB: cAMP response element-binding protein
TET: ten-eleven translocation
5hmC: 5-hydroxymethylcytosine
PAR-2: protease-activated receptor 2
HGF: hepatocyte growth factor
SDF-1/CXCL12: stromal-derived factor-1
